# Product formulation and rubbing time impact the inactivation of enveloped and non-enveloped virus surrogates by foam-based hand sanitizers

**DOI:** 10.1128/aem.02474-24

**Published:** 2025-03-25

**Authors:** Francis Torko, Kristen E. Gibson

**Affiliations:** 1 Department of Food Science, Center for Food Safety, University of Arkansas System Division of Agriculture 3341 , Fayetteville, Arkansas, USA; Centers for Disease Control and Prevention, Atlanta, Georgia, USA

**Keywords:** hand hygiene, formulation, rubbing time, foam hand sanitizer, efficacy

## Abstract

Effective hand hygiene, such as hand washing and hand sanitizer use, is crucial in reducing infectious disease transmission via the hands. The efficacy of hand washing has been well-documented; however, relatively less is known regarding foam-based hand sanitizer efficacy, which is considered an effective alternative to washing hands with soap and water. Hand sanitizers are recommended by both the United States Centers for Disease Control and Prevention and the World Health Organization when the hands are not visibly dirty or greasy. This study examined the efficacy of five commercially available foam hand sanitizers—four alcohol-based and one non-alcohol-based—against enveloped and non-enveloped viruses using bacteriophage phi6 (Φ6) and bacteriophage MS2 as surrogates, respectively. A cocktail of MS2 and Φ6 (8 log PFU/mL) was inoculated on the hands and exposed to 3 or 6 mL of hand sanitizer product followed by rubbing the palmar surface of the hands together for 10 s or until dry. The results showed significant log reduction among the virus surrogates (*P* ≤ 0.05), with Φ6 consistently showing higher susceptibility across all factors compared with MS2 with log reductions of 2.83 ± 1.98 and 0.50 ± 0.53 log reduction, respectively. Although dosing volume did not significantly impact log reduction (*P* = 0.31), rubbing time significantly affected bacteriophage inactivation (*P* ≤ 0.05). Higher log reduction was observed when hands were rubbed until dry (2.69 ± 2.06), compared with the typical 10 s rubbing time (0.65 ± 0.75). This study revealed that the efficacy of commercially available foam hand sanitizers depends on rubbing time and overall product formulation, rather than exclusively on active ingredient concentration.

**IMPORTANCE:**

Human hands are a key factor in the transmission of viral diseases, and proper hand hygiene is regarded as the gold standard against the spread of such diseases. This study examined the effectiveness of a hand hygiene technique, that is, the application of foam-based hand sanitizers, against the inactivation of enveloped and non-enveloped virus surrogates on the hands. Factors such as virus type, rubbing time, volume of product used, and product formulation can significantly influence the efficacy of hand sanitizers. To assess these effects, we tested different rubbing times and product volumes across alcohol- and non-alcohol-based, foam hand sanitizer formulations, each with varying active ingredient concentrations and inactive ingredients. The study was performed on the palmar surface of human hands to realistically simulate real-world conditions, providing valuable evidence to inform future hand sanitizer practices aimed at maximizing the reduction of infectious viral pathogens on the hands.

## INTRODUCTION

Effective hand hygiene is a critical step in reducing the transfer of pathogens between persons and fomites (1). According to the United States Centers for Disease Control and Prevention (CDC), hand hygiene involves cleaning the hands to substantially reduce the amount of potentially harmful microorganisms that might be present on the hands. This can be achieved through hand washing with soap under running water or using hand sanitizers. Hand washing has been repeatedly shown to effectively reduce the amount of pathogens on the hands (2–4). Godoy et al. (2) established that washing hands at least five times a day or after touching contaminated fomites significantly protected against influenza A (H1N1). Similarly, a systematic review by Xun et al. (4) led to the conclusion that increased frequency of hand washing lowers the risk of acquiring diseases. Hand washing is the preferred form of hand hygiene when the hands are visibly dirty or greasy; however, in the absence of soap and water, the use of a hand sanitizer is the alternative form of hand hygiene recommended by both the CDC and the World Health Organization (WHO). However, although there are numerous studies on the efficacy of hand hygiene techniques in the control of pathogens, particularly around hand washing, comparatively little is known about the efficacy of commercially available hand sanitizers, especially as formulations and formats have evolved over time.

The CDC recommends that alcohol-based hand sanitizers (ABHS) contain a minimum of 60% alcohol (5), whereas the WHO recommends 80% ethanol or 75% vol/vol isopropanol (6). Although most previous studies noted the concentration of active ingredients as a key factor in hand sanitizer efficacy (7, 8), recent publications suggest that the efficacy of hand sanitizers may not solely depend on the concentration of active ingredients but rather on the overall formulation of the product (9–12). Escudero-Abarca et al. (9) investigated the efficacy of one non-alcohol-based hand sanitizer (NABHS) with benzalkonium chloride as the active ingredient and seven ABHSs with ethanol as the active ingredient, which included foam, gel, and liquid formats, against human norovirus (HuNoV) GII.4 on fingerpads. The authors found that the efficacy of the hand sanitizers was not exclusively dependent on the active ingredients but on the product formulations. This conclusion was based on observing statistically significant differences between products with similar active ingredient concentrations (85% vol/vol and 80% wt/wt [85 vol/vol] ethanol) as well as a lack of significant differences between products with varying active ingredient concentrations (85 vol/vol compared with 68%–70% vol/vol ethanol). This observation is supported by Siddharta et al. (11) and Suchomel et al. (12) in their exploration of the efficacy of modified WHO formulations where reduced glycerol—an inactive ingredient—appeared to positively impact the efficacy of the formulations.

In environments with high contact rates, such as childcare centers, food establishments, public transport, and gyms, contaminated surfaces and hands play a critical role in pathogen transmission (13). Both enveloped viruses [e.g., influenza and coronaviruses, including severe acute respiratory syndrome coronavirus-2 (SARS CoV-2)] and non-enveloped viruses (e.g., HuNoV) can potentially be transmitted via contaminated surfaces (14–18). Bloomfield et al. (13) also further investigated the role of hands and secondary surfaces as critical points for the spread of infectious diseases. The authors noted that hands often make primary contact with pathogen points of entry such as the eyes, mouth, and nose, highlighting the role of effective hand hygiene in controlling the spread of diseases such as gastrointestinal and respiratory infections.

As previous studies suggest, the efficacy of hand sanitizers can be influenced by several factors, including active ingredient concentration, exposure/rubbing time, dosing volume, and overall formulation. However, many of these studies face limitations, such as the lack of direct comparisons between different rubbing times and dosing volumes for both enveloped and non-enveloped virus inactivation under similar testing conditions. Variations in methodologies across studies make it challenging to compare efficacy results between virus types, as testing methods have been shown to significantly impact estimates of microbial reduction (19). Moreover, past research often focused on active ingredient concentrations in lab-based solutions rather than in commercially available hand sanitizer formulations (7, 20). Furthermore, although data on foam hand sanitizers’ efficacy against viruses is limited, most research has been conducted in suspension, with *in vivo* studies primarily performed on fingerpads—an approach less representative of real-world use compared with the whole hand.

This study aimed to address these gaps by comparing five foam-based hand sanitizers for the inactivation of enveloped and non-enveloped viruses on hands, using bacteriophages—viruses that specifically infect and propagate in bacterial cells (21)—Φ6 and MS2 as surrogates. More specifically, an assessment of dosing volumes, rubbing times, and overall formulation was completed to determine the impact on the efficacy of ABHS and NABHS. Φ6 was selected as a surrogate to study enveloped viruses based on its prior use in hand-related research and recommendations from previous studies (22–24). Similarly, MS2 is a well-established surrogate for the study of non-enveloped viruses (8, 25). The outcome of this research will contribute valuable insights to inform effective hand sanitizer usage strategies to control infectious disease transmission.

## MATERIALS AND METHODS

### Volunteer recruitment

Thirty volunteers (≥18 years old) gave their consent to participate in this *in vivo* hand hygiene efficacy study. All volunteers had no skin diseases or any apparent damage such as dermatitis, open wounds, cuts, burns, hung nails, or any additional known hand disorders. The University of Arkansas Institutional Review Board approved all protocols used here (Protocol number: 2009287687R002). Five commercially available hand sanitizers, including one NABHS and four ABHS, were investigated for their efficacy against enveloped and non-enveloped virus surrogates according to the American Society for Testing and Materials (ASTM) E2011-21 (26) with modifications.

### Phage propagation and quantification

Bacteriophages Φ6 (HER102; procured from Dr. Sylvain Moineau at Université Laval in Québec, Canada) and MS2 [American Type Culture Collection (ATCC) 15597-B1, Manassas, VA] were used as surrogates for the study of enveloped and non-enveloped viruses, respectively. Using previously described methods (22, 27), *Pseudomonas syringae pathovar phaseolicola* (Pph; HER1102) was used as a host for Φ6 propagation on lysogeny agar/broth (LC; 10 g/L NaCl, 10 g/L tryptone, 5 g/L yeast extract, pH 7.5), whereas *Escherichia coli* C3000 (ATCC 15597) was used as a host for MS2 propagation on tryptic soy broth/agar (TSB/TSA) (30 g/L of TSB; Becton, Dickson and Company, Sparks, Maryland). A single colony of Pph or *E. coli* was cultured overnight in 25 mL of LC broth or TSB in a shaking incubator at 125 rpm. One hundred microliters of overnight *E. coli* culture was added to 15 mL of TSB and put in the shaking incubator for 3 to 4 h to attain a log phase growth prior to assay. Using the double agar overlay (DAL) assay, Φ6 and MS2 were propagated by mixing 250 µL of Pph overnight liquid culture or 50 µL of *E. coli* overnight log phase growth culture with 100 µL of Φ6 or MS2 stock (at approximately 9 or 10 log PFU/mL) in 5 mL of LC or TSA soft agar, respectively. The mixture was then poured onto LC or TSA plates and incubated at 25°C (Φ6) or 37°C (MS2). Plates that were observed to show lysis of host cells were harvested by scrapping the soft agar overlay with a 25 cm cell culture scraper (VWR, Radnor, PA) into a 50 mL centrifuge tube. The underlying hard agar was rinsed with 4 mL of LC (Φ6) or TSB (MS2), and the broth was transferred to the 50 mL centrifuge tube. The mixture was vortexed for 15 s and centrifuged for 10 min at 2000 × *g* at 4°C. The supernatant was then filtered through a 0.22 µm, 33 mm diameter polyvinylidene fluoride (PVDF) membrane filter (EZFlow Syringe Filter; Foxx Life Sciences, Londonberry, NH), and stored at −20°C (Φ6) and −80°C (MS2) for later use. Quantification of viruses was performed by serially diluting the propagated phages, followed by the DAL method as previously described (22, 27). Phage stocks were prepared using DAL (as described here) and stored at −20°C for Φ6 and −80°C for MS2 prior to use for propagation. Additionally, no serial dilutions were performed prior to the use of phage stocks for Φ6 and MS2 propagation.

### Organic matter and virus cocktail preparation

The ASTM tripartite organic matter solution was prepared following the ASTM E2011-21 guidelines as a source of organic matter (OM). Specifically, 0.5 g of yeast extract, 0.5 g of bovine serum albumin (BSA), and 0.04 g of bovine mucin were each separately added to 10 mL of 1× PBS and subsequently filtered via a 0.22 µm, 33 mm diameter PVDF membrane filter (EZFlow Syringe Filter; Foxx Life Sciences). A virus cocktail comprising Φ6 and MS2 was prepared by suspending both phages in LC broth to achieve a final concentration of 8 log plaque forming units (PFU)/mL. The final cocktail suspension included the tripartite solution consisting of 20% bovine mucin, 7% yeast extract, and 5% BSA. Tripartite stocks were stored at 4°C until use.

### Volunteer hand preparation

Hand preparation was performed using previously described methods (22). Volunteers were instructed to wash their hands with non-antimicrobial soap (Equate Hand Soap; Walmart Inc., Bentonville, Arkansas), for 60 s (30 s wash with soap and 30 s rinse with water) under running tap water and dried thoroughly with paper towels. Approximately 5 mL of 80% ethanol was applied to the hands, and volunteers were instructed to rub the ethanol over the entire hands until dry. Hands were then rinsed with approximately 200 mL of sterile deionized water and dried with paper towels. Volunteers were instructed to avoid contact with all surfaces.

### Hand inoculation and hand sanitizer treatment

Inoculation of volunteers’ hands was performed according to ASTM E2011-21 with modifications. One milliliter of the cocktail virus suspension (~8 log PFU/mL of each virus), in the presence of ASTM tripartite organic matter, was inoculated onto the palm of the volunteer’s left hand, and using their right hand, the inoculum was distributed over the palmar surface of both hands for 10 s while avoiding contamination of the backs of hands or dripping of the inoculum. Following hand inoculation, one unit (3 mL foam, equivalent to ~0.4 mL liquid) or two units (6 mL foam, equivalent to ~0.8 mL liquid) of foam hand sanitizer (Table 1) was dispensed, using an automatic dispenser (Alpine Hand Sanitizer Dispenser; Alpine Industries Inc., Irvington, New Jersey), onto the palm of the volunteer’s left hand. The sanitizer was then distributed over the palmar surfaces of both hands using the right hand for 10 ± 1 s or until dry (range from 2.12 min to 6.42 min), and any remaining virus on the hands was recovered as described in the “Virus Recovery” section. 10 ± 1 s rubbing time was based on preliminary studies on how long it typically takes users to rub a hand sanitizer product (data not shown).

**TABLE 1:** Foam-based hand sanitizers investigated and their formulations^*a*^

Product	Brand name	Formulation
A	Global industrial foam hand sanitizer 62% alcohol	***62% ethanol***, H_2_O, PEG-10 dimethicone, glycerin, isopropyl myristate, polyquaternium-11, disodium EDTA, aloe barbadensis leaf juice, tocopheryl acetate
B	Global industrial foam hand sanitizer alcohol-free	***0.13% BZK***, H_2_O, propylene glycol, cocamidopropyl betaine, aloe barbadensis leaf juice, tocopheryl acetate (vitamin E), PEG-7 glyceryl cocoate, fragrance, phenoxyethanol, tetrasodium EDTA
C	PURELL advanced hand sanitizer green certified foam	***70% ethanol***, H_2_O, isopropyl alcohol, PEG-12 dimethicone, caprylyl glycol, glycerin, isopropyl myristate, tocopheryl acetate
D	Tork premium alcohol foam hand sanitizer	***70% ethanol***, H_2_O, glycerin, carbomer, glycereth-7 triacetate, triethanolamine, tocopheryl acetate
E	SCJ professional hand sanitizer, non-alcohol	***85% ethanol***, H_2_O, DL-panthenol, Bis-PEG-12 dimethicone, dihydroxypropyl PEG-5 linoleammonium chloride, PEG-200 hydrogenated, glyceryl palmitate, PEG-7 glyceryl cocoate, coco-glucoside, glyceryl oleate, citric acid

^*a*^ BZK, benzalkonium chloride; bold and italics indicate the active ingredient.

### Virus recovery and quantification

Virus recovery was performed using a modification of the dish method according to the ASTM E1838-17 (28) protocol based on preliminary studies for recovery optimization (29). After hand sanitizer treatment, the volunteer pressed both palms onto the bottom of a sterile dish container (Autoclavable Polypropylene Pan, 12.75 × 4.25 × 10.125 in [L × H × W]; Fisher Scientific, Rochester, NY) containing 3.5 mL of D/E neutralizing broth and 66.5 mL of LC broth (1:20) and rubbed continuously for 1 min ± 5 s. After recovery, 70% ethanol was then sprayed onto the volunteer’s hands and rubbed over the entire hand. Hands were then washed thoroughly using plain soap and water followed by drying with a paper towel. The recovered eluate was transferred into a sterile container (Media/Storage Bottle, 100/150 mL, VWR, Radnor, PA) and assayed to determine the concentration of the recovered virus using the DAL method. Briefly, a 5-fold or 2-fold serial dilution was performed using LC broth as diluent. One hundred microliters of serially diluted samples were then added to 5 mL of TSA or LC soft agar media containing 50 µL of *E. coli* or 250 µL of Pph. The mixture was added to TSA or LC plates and incubated overnight at 25°C (Φ6) or 37°C (MS2). Control plates were prepared to determine the potential host or broth contamination and cytopathic effect of phages. Host control consisted of 50 µL of *E. coli* or 250 µL of Pph in 5 mL of TSA or LC soft agar. For virus control, 100 µL of highly concentrated (approximately 8 log PFU/mL) MS2 or Φ6 was added to their respective host and soft agar. Broth control included 100 µL of TSB or LC broth in 5 mL of TSA or LC soft agar.

### Neutralizer effectiveness/toxicity

Neutralization of test products was conducted during the recovery of phages from hands, and the determination of neutralization effectiveness and cytotoxicity was performed following ASTM E1054-22 (30). The neutralizer-nutrient medium was prepared at a final ratio of 1:20 using D/E (Dey/Engley) Neutralizing media (BD Life Sciences, Franklin Lakes, New Jersey) and LC broth. Briefly, a 100 µL cocktail of Φ6 and MS2, in the presence of ASTM tripartite organic matter, containing between 10^3^ and 10^4^ virus particles was introduced into 10 mL of the neutralizing media and LC broth mixture at a 1:20 ratio (0.5 mL neutralizer + 9.5 mL LC broth). To evaluate the neutralizer’s effectiveness, 114 µL of hand sanitizer product was added to the neutralizer-LC-virus suspension, which corresponds to the scaled highest volume of liquid hand sanitizer used in the study. For the cytotoxicity test, 114 µL of 1× PBS was added to the neutralizer-LC-virus suspension instead of the hand sanitizer product. As a control, the same volume of virus suspension was added to 10.114 mL of LC. The resulting mixture was tested to determine the phage concentration and potential impact on the DAL assay. The neutralization assay results showed no significant differences in neutralization effectiveness, cytotoxicity, and control for Φ6 (*P* = 0.75) and MS2 (*P* = 0.19). Comparable results were also observed among individual hand sanitizers with effectiveness, cytotoxicity, and control.

### Statistical analysis

All statistical analyses were performed using the R statistical software version 4.2.2. A generalized linear mixed effects model (“Gamma” family and “log” link) was developed to assess the statistical significance between the levels of fixed factors (virus type, product, dosing volume, rubbing time), estimate variance for random factors (volunteers), and determine significant two-way interactions between the variables. Since the Gamma distribution with a log link cannot take 0 values, a constant of 0.5 was added to all log reduction values to determine statistical significance without impacting the statistical analysis. The model’s normality and homoskedasticity of variance assumptions were examined using the Shapiro-Wilk and Breusch-Pagan tests, respectively. The results indicated that assumptions for both normality (*P* = 0.16) and homoskedasticity (*P* = 0.98) were met. Type 3 analysis of variance of the generalized linear mixed effects model was used to detect statistical significance at *P* ≤ 0.05. Volunteers were randomly assigned to a hand sanitizer product and dosing volume unit but received both treatment levels of rubbing time on separate days, with at least 1 week between treatments, in a randomized order. All experimental units were performed in triplicate. The amount of virus suspended on the hands was calculated by performing a DAL assay of the cocktail inoculum suspension, and the expected amount to be recovered was determined by dividing the suspended amount of PFU by 70 mL. Plaque-forming unit values were log_10_ transformed to log PFU/mL for subsequent analyses. As a treatment control, the expected value was multiplied by a factor of 1 for MS2 and by a factor of 0.95 for Φ6 as preliminary results (data not shown) showed that about 100% and 95% recovery efficiencies from hands were observed for MS2 and Φ6, respectively. Treatment values were the amount of virus recovered after the hand sanitizer treatment, and log reduction (response factor) was calculated by determining the difference between the treatment and control values. All negative log reduction values (*n* = 11; range = [-0.2 to −0.02]), which occurred when treatment values exceeded control values, were assigned a 0 to indicate no log reduction or no product efficacy. For treatment, replicates where no PFU was detected, 1 PFU/mL (limit of detection) was assigned in order to log transform the data to calculate the log reduction achieved by the test products. Efficacy of the hand sanitizer products, expressed in percentage, for Φ6 and MS2 across all treatment combinations can be found in the supplemental material.

## RESULTS AND DISCUSSION

Previous studies have explored the efficacy of hand sanitizers for the reduction of infectious virus particles, often focusing on correlating hand sanitizer efficacy to an increasing concentration of active ingredients (7, 8, 25). However, in the present study, the efficacy of the tested foam hand sanitizers against both enveloped and non-enveloped phage did not correlate with increasing concentrations of the active ingredients, consistent with findings from previous studies (9–12, 31). Instead, these findings suggest that foam hand sanitizer efficacy is influenced in part by the overall formulation, including inactive ingredients.

For instance, across all levels of factors, the average highest log reduction of 2.06 ± 2.22 was achieved by product D (70% vol/vol ethanol) followed by products B (0.13% BZK), C (70% vol/vol ethanol), E (85% wt/wt ethanol), and A (62% vol/vol ethanol) (Table 2). Although product D had the highest log reduction, its efficacy was comparable with products B, C, and E. Moreover, product E had a comparable log reduction with products B, C, and D, as well as product A, which had the lowest log reduction. Similarly, Escudero-Abarca et al. (9) observed significant differences in reduction of HuNoV GII.4 among products that had similar active ingredient concentrations [85% vol/vol and 80% wt/wt (85 vol/vol) ethanol], whereas products with varying active ingredient concentrations (85% vol/vol compared with 68%–70% vol/vol ethanol) did not show any significant differences as observed in the current study. Macinga et al. (10) also studied the efficacy of hand sanitizer formulations with different active ingredients against native microorganisms on hands after single and repeated use. Results showed that the highest log reduction (4.37) was achieved with a 70% ethanol-based hand rub, which was superior to a 62% ethanol-based product (1.86 log). However, a 63% 2-propanol-based product demonstrated a higher log reduction than a 70% 2-propanol product formulated differently, emphasizing that overall product formulation impacts efficacy. The results from this study underscore that hand sanitizer efficacy is affected by overall product formulation.

**TABLE 2:** Efficacy of foam-based hand sanitizer products against MS2 and φ6 across all variables^*a*^^,^^*b*^

Product	Log reduction^*c*^	
	Phi 6	MS2
A (62% EtOH)	2.33 ± 1.53^a^	0.92 ± 0.43^a^
B (0.13% BZK)	3.94 ± 2.12^c^	0.95 ± 0.43^a^
C (70% EtOH)	3.51 ± 2.01^bc^	1.13 ± 0.84^a^
D (70% EtOH)	4.08 ± 2.24^c^	1.03 ± 0.49^a^
E (85% EtOH)	2.79 ± 1.61^ab^	0.97 ± 0.35^a^

^*a*^ Efficacy is expressed as log reduction in virus concentration (log PFU/mL).

^*b*^ BZK, benzalkonium chloride; EtOH, ethanol.

^*c*^ Log reduction values represent arithmetic mean log reduction across all variables ± standard deviation, and statistical significance was determined using Tukey’s honestly significant difference test. Values sharing the same superscript letters are not significantly different.

Although these findings emphasize the importance of formulation on hand sanitizer efficacy, earlier studies primarily focused on the role of active ingredient concentration as mentioned early on. For example, Park and coauthors (8) investigated the *in vitro* efficacy of seven hand sanitizers, four of which contained ethanol as an active ingredient. The results showed that three of the hand sanitizers with higher ethanol concentrations (72%–79%) exhibited rapid inactivation of murine norovirus (MNV, a HuNoV surrogate) by more than 2.6-log to 3.6-log reduction after 1 min in suspension. In comparison, one of the products with 67% ethanol achieved only a 2.0 log reduction after 1 min, indicating that higher active ingredient concentration (ethanol in this case) increases efficacy. Similarly, Tung et al. (25) established that MNV exhibited higher susceptibility to ethanol at elevated ethanol concentrations (70 and 90% compared with 50%), reinforcing the idea that increasing concentration enhances inactivation. However, the findings of the current study, along with those of previous research, indicate that the efficacy of hand sanitizers may not only depend on the active ingredients but also on overall hand sanitizer formulation.

**Fig 1:**
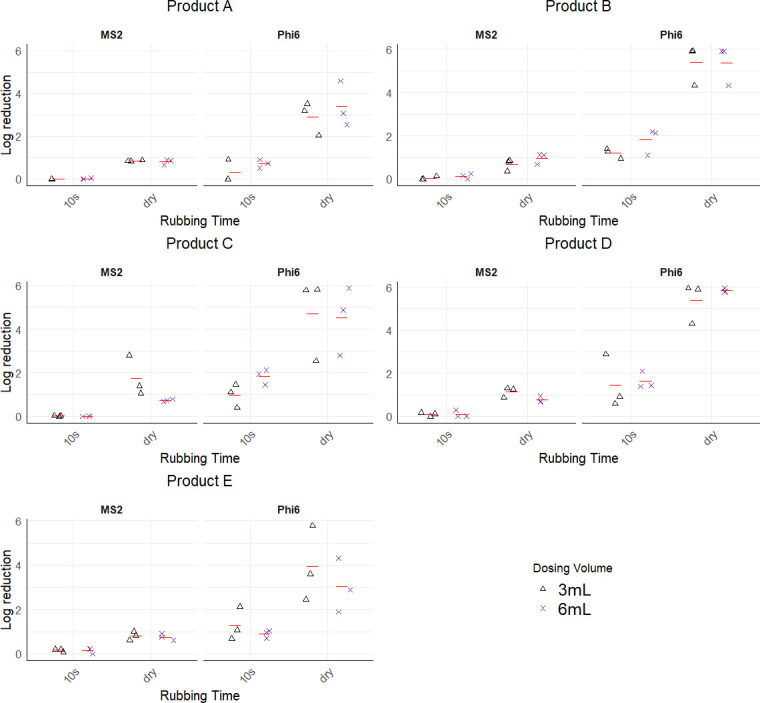
Efficacy of hand sanitizer products against Φ6 (*Pseudomonas* phage Φ6) and MS2 (*Emesvirus zinderi*) for 10 s and “until dry” rubbing times for 3 mL and 6 mL dosing volumes. Each data point represents the difference between treatment and control values. The red line represents the mean of the factor levels. The open triangle and cross symbols represent 3 mL and 6 mL dosing volumes, respectively. Log reduction values were expressed in log PFU/mL.

Although some studies found that BZK-based hand sanitizers perform poorly against non-enveloped viruses when compared with ethanol-based products or solutions (9, 10), others demonstrated that BZK performs better (20). For instance, in a fingerpad study by Wilson et al. (20), a BZK containing liquid hand sanitizer achieved 2.13 ± 0.50 and 2.09 ± 0.35 log reduction of HuNoV GII.4 after 30 s and 60 s exposure times, respectively, compared with a benchmark 60% ethanol solution, which achieved log reduction of 1.06 ± 0.54 and 1.22 ± 0.56 for the same exposure times. In this study, the BZK-based hand sanitizer (0.13% BZK) had a significantly higher (*P* ≤ 0.05) log reduction than product A (62% vol/vol ethanol) similar to Wilson et al. (20) but was comparable with products C, D (both containing 70% vol/vol ethanol), and E (85% wt/wt ethanol) across both phages (Fig. 1).

In addition to active ingredient concentration and hand sanitizer formulation, exposure/rubbing time and product dosing volume may also be relevant for virus inactivation (7, 9, 32, 33). Most of these studies were conducted *in vitro* with a limited number of *in vivo* studies performed primarily on fingerpads (3, 9, 20, 34–36). Meanwhile, the present study also investigated the impact of rubbing time and hand sanitizer dosing volume on the efficacy of foam-based hand sanitizers against both enveloped and non-enveloped virus surrogates on hands. Table S1 (in the supplemental material) reports the percent efficacy of each product based on the variables of rubbing time, dosing volume, and virus type.

Non-enveloped viruses are generally less susceptible to inactivation with chemical sanitizers (7, 37, 38) due to the high stability of their protein capsids (39). The observed virucidal activity against the enveloped virus surrogate (bacteriophage Φ6) and non-enveloped virus surrogate (bacteriophage MS2) is consistent with a review by Kampf (7) on the efficacy of ethanol in solution against enveloped and non-enveloped viruses where it was found that enveloped viruses were generally less resistant than non-enveloped viruses. Here, it was observed that, across factors, the average log reduction achieved for Φ6 (2.83 ± 1.98) was significantly higher (*P* ≤ 0.05) than MS2 (0.5 ± 0.53). Likewise, Van Engelenburg et al. (38) found that an ethanol-based hand rub mixture completely inactivated all tested enveloped viruses (more than a 6 log reduction) including hepatitis C, whereas non-enveloped viruses, including hepatitis A virus, were more resistant with log reduction ranging from no reduction to a maximum of 6 log.

Beyond virus structure, exposure time plays a crucial role in virus activation, with longer contact time between hand sanitizers and viruses generally linked to greater virus inactivation (20, 40). In a study by Kampf et al. (40), although results were not directly investigated for different exposure times for the same virus, it was evident that higher exposure times were required for ABHSs to achieve similar log reductions across various non-enveloped viruses. Although other factors, such as varying stability of viral protein capsid, may have contributed to reduced susceptibility of viruses in Kampf et al. (40), exposure time was clearly an influential factor. For instance, whereas 2- and 3-min exposure times were needed to achieve >4 log reduction for adenovirus and poliovirus, respectively, papovavirus required 15 min. Similarly, in a study by Wilson et al. (20), which compared a BZK-based hand sanitizer and a 60% ethanol solution on fingerpads, the authors reported that the highest log reduction for both products was achieved at 60 s exposure time compared with a 30 s exposure time. The findings from the current study align with Wilson et al. (20). Rubbing the hands until dry (range from 2.12 min to 6.42 min) after sanitizer application demonstrated greater efficacy compared with a 10 s rub (Fig. 1; Table S1). A 10 s rubbing time consistently led to significantly lower (*P* ≤ 0.05) log reduction (0.65 ± 0.75) than until dry (2.69 ± 2.06) for phages combined (Fig. 1). This indicates that rubbing the hands until dry after applying a foam hand sanitizer achieves increased virus inactivation on the hands than the typical 10 s rubbing time, reinforcing the importance of extended contact time for effective virus reduction. Regarding dosing volume, no significant differences (*P* = 0.31) were observed in log reduction for 3 mL (1.66 ± 1.89) and 6 mL (1.67 ± 1.84) dosing volumes.

To better understand the efficacy of hand sanitizers against individual phages, we compared phage susceptibility across the tested factors. Among products, significant differences (*P* ≤ 0.05) were observed in log reduction for Φ6 but no significant differences (*P* = 0.13) were observed for MS2 (Table 2). Significant differences (*P* ≤ 0.05) were observed in rubbing time for both Φ6 and MS2. The “until dry” rubbing time yielded significantly higher (*P* ≤ 0.05) log reduction (0.93 ± 0.42 for MS2 and 4.45 ± 1.44 for Φ6) than the typical 10 s rubbing time (0.07 ± 0.1 for MS2 and 1.22 ± 0.68 for Φ6), with Φ6 consistently showing higher log reduction than MS2. Dosing volume did not significantly impact log reduction for Φ6 (*P* = 0.07) and MS2 (*P* = 0.55).

For MS2, significant two-way interactions were found between product and volume, product vs rubbing time, and volume vs rubbing time (*P* ≤ 0.05). This suggests that log reduction achieved with each product depends on both dosing volume and rubbing time. Although the dosing volume alone did not significantly differ, the 3 mL volume yielded a higher log reduction than 6 mL. Also, log reductions of MS2 for the dosing volumes were dependent on rubbing time because, while “until dry” achieved greater reduction regardless of the dosing volume applied to the hands, 3 mL had a significantly higher log reduction than 6 mL when paired with “until dry” rubbing time.

For Φ6, a significant interaction (*P* ≤ 0.05) was observed between product and rubbing time as well as volume and rubbing time indicating that product efficacy depends on the rubbing time and dosing volume. During both 10 s and until dry rubbing times for Φ6, product A recorded the lowest significant log reduction whereas products B, C, D, and E had comparable log reductions. Although product A had the lowest log reduction, its efficacy was comparable with product E when a 10 s rubbing time was used and products B, C, and E when hands were rubbed until dry. Although no significant log reduction (*P* = 0.97) was observed when hands were rubbed until dry, 6 mL dosing volume had a significantly higher (*P* ≤ 0.05) log reduction than 3 mL dosing volume when a 10 s rubbing time was employed.

Although this study closely reflects real-world scenarios, it has some limitations. Despite appropriate measures observed to apply inoculum solely to the palmar surface, the potential contamination of the back of the hand and dripping could have influenced virus quantification. However, when this was observed, the hand preparation and inoculum procedures were repeated prior to hand sanitizer treatment to ensure consistent inoculum application. Future studies should consider using smaller inoculum volumes to better accommodate volunteers with smaller palmar surface areas. In addition, although the inoculum was still wet before sanitizer application, minimizing the effect of drying on virus inactivation, the authors acknowledge that drying may impact virus quantification, particularly with “until dry” rubbing time. Future studies should examine the effect of drying on virus inactivation when investigating an “until dry” rubbing/exposure time. Hand characteristics, such as the size of the palm or texture of the skin, could also have affected factors such as until dry rubbing time or inoculum spillage, although the latter was controlled as indicated previously. To estimate variation due to individual volunteers, volunteer variability was estimated, using a generalized linear mixed effects model, revealing that approximately 15% of the variation in virus log reduction was due to individual differences. This suggests that volunteer characteristics, including palm size, texture, or until dry rubbing time, contribute minimally to moderate random variation to the results.

Overall, the results from this study indicate that foam hand sanitizer efficacy against viruses is influenced by the product formulation, rubbing time, and virus type. This suggests that manufacturers should focus on complete formulation instead of active ingredient concentration, as the latter does not fully dictate antiviral activity. Additionally, virus type also plays a role, with enveloped viruses being more susceptible than non-enveloped viruses (Fig. 1). To maximize viral reduction, hands should be rubbed until dry. Testing on the palmar surface in this study enhances real-world relevance, as this area of the hand is primarily in contact with surfaces as well as direct contact between individuals. Finally, two-way interaction results revealed that the efficacy against non-enveloped viruses may be affected by a wider range of factors compared with enveloped viruses. To the best of our knowledge, this is the first *in vivo* study to comparatively investigate the impact of rubbing time, dosing volume, and formulation on the efficacy of foam-based hand sanitizers against both enveloped and non-enveloped virus surrogates utilizing the palmar surface—an approach that closely reflects real-world scenarios.

## SUPPLEMENTAL MATERIAL

Table S1 aem.02474-24-s0001.docx Efficacy in percentages of foam hand sanitizer products for bacteriophages Φ6 and MS2 across all factor levels.

ASM does not own the copyrights to Supplemental Material that may be linked to, or accessed through, an article. The authors have granted ASM a non-exclusive, world-wide license to publish the Supplemental Material files. Please contact the corresponding author directly for reuse.
