# Supplementary material for: Product formulation and rubbing time impact the inactivation of enveloped and non-enveloped virus surrogates by foam-based hand sanitizers
Source: Appl Environ Microbiol. 2025 Mar 25;91(4):e02474-24. doi: 10.1128/aem.02474-24 (PMC12016521; doi:10.1128/aem.02474-24)
Supplement: Table S1 — Efficacy in percentages of foam hand sanitizer products for bacteriophages Φ6 and MS2 across all factor levels. [file aem.02474-24-s0001.docx]

**Supplemental Table S1:** Efficacy in percentages of foam hand sanitizer products for bacteriophages Φ6 and MS2 across all factor levels. Values represent arithmetic mean ± standard deviations.

| **Product** | **Volume** | **Rubbing Time** | **Virus Surrogate Type** | |
| --- | --- | --- | --- | --- |
|  |  |  | MS2 | Φ6 |
| A | 3 mL | 10 s | 0.759 ± 1.314 | 29.307 ± 50.762 |
|  |  | Until dry | 85.967 ± 0.808 | 99.657 ± 0.511 |
|  | 6 mL | 10 s | 2.933 ± 5.080 | 79.323 ± 9.444 |
|  |  | Until dry | 83.535 ± 5.166 | 99.875 ± 0.148 |
|  |  |  |  |  |
| B | 3 mL | 10 s | 8.623 ± 14.935 | 93.191 ± 3.764 |
|  |  | Until dry | 75.685 ± 16.768 | 99.998 ± 0.003 |
|  | 6 mL | 10 s | 23.887 ± 21.722 | 96.836 ± 4.283 |
|  |  | Until dry | 87.855 ± 7.585 | 99.998 ± 0.003 |
|  |  |  |  |  |
| C | 3 mL | 10 s | 5.866 ± 5.08 | 83.17 ± 19.471 |
|  |  | Until dry | 95.686 ± 4.281 | 99.903 ± 0.167 |
|  | 6 mL | 10 s | 2.225 ± 3.854 | 98.183 ± 1.51 |
|  |  | Until dry | 81.564 ± 2.347 | 99.946 ± 0.092 |
|  |  |  |  |  |
| D | 3 mL | 10 s | 18.769 ± 17.250 | 87.267 ± 12.837 |
|  |  | Until dry | 92.039 ± 4.797 | 99.998 ± 0.003 |
|  | 6 mL | 10 s | 16.997 ± 27.491 | 97.167 ± 1.78 |
|  |  | Until dry | 82.403 ± 5.302 | 99.999 ± 2.89 |
|  |  |  |  |  |
| E | 3 mL | 10 s | 31.321 ± 10.939 | 90.166 ± 9.889 |
|  |  | Until dry | 83.816 ± 7.21 | 99.873 ± 0.198 |
|  | 6 mL | 10 s | 26.953 ± 23.352 | 86.836 ± 5.524 |
|  |  | Until dry | 81.975 ± 5.847 | 99.523 ± 0.713 |

Φ6, bacteriophage Phi 6; MS2, *Emesvirus zinderi*
